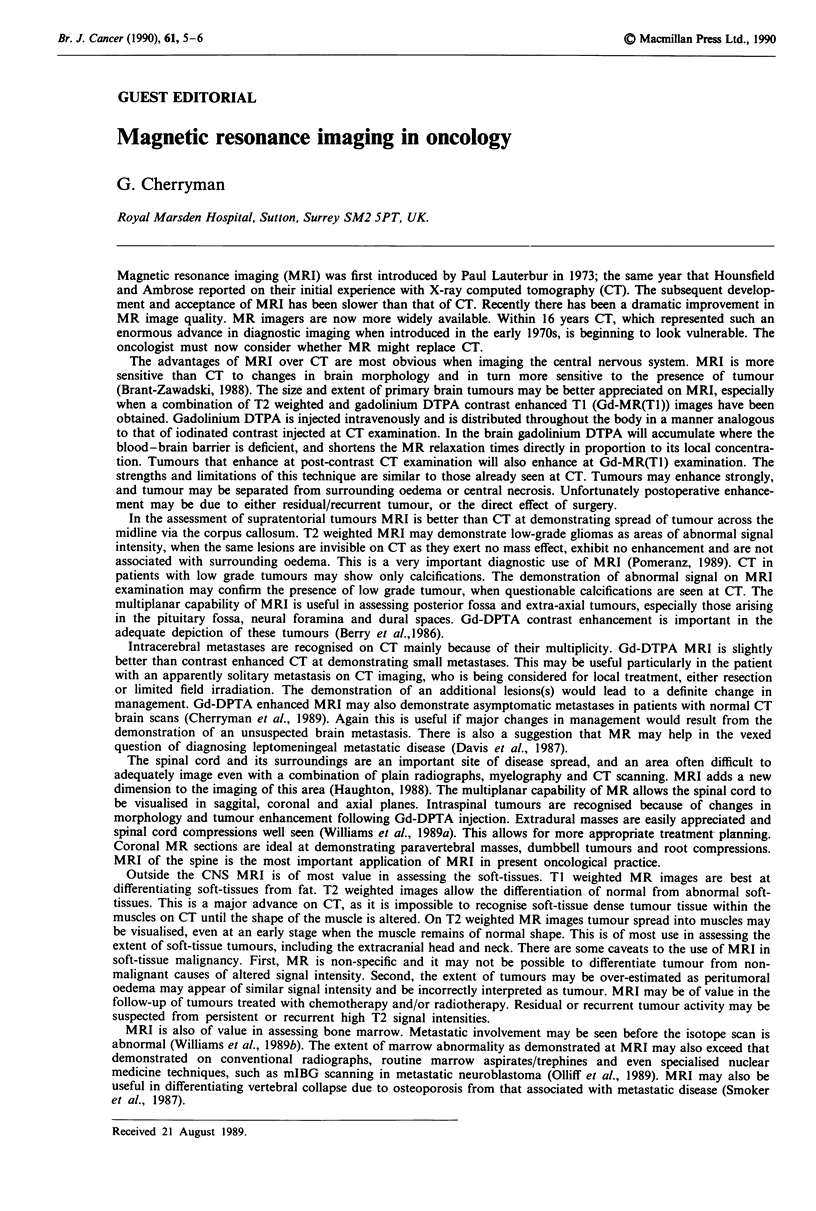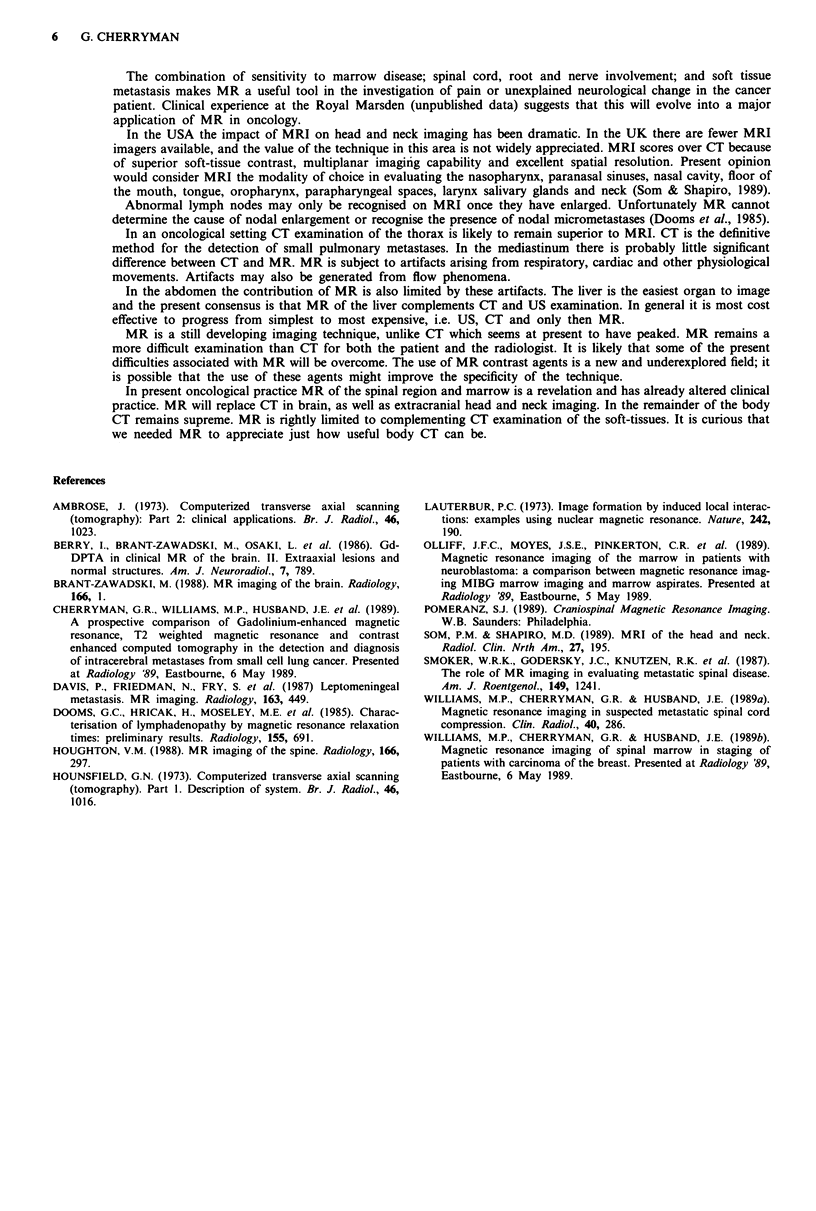# Magnetic resonance imaging in oncology.

**DOI:** 10.1038/bjc.1990.2

**Published:** 1990-01

**Authors:** G. Cherryman

**Affiliations:** Royal Marsden Hospital, Sutton, Surrey, UK.


© Macmillan Press Ltd., 1990

GUEST EDITORIAL

### Magnetic resonance imaging in oncology

G. Cherryman

*Royal Marsden Hospital, Sutton, Surrey SM2 5PT, UK.*

Magnetic resonance imaging (MRI) was first introduced by Paul Lauterbur in 1973; the same year that Hounsfield and Ambrose reported on their initial experience with X-ray computed tomography (CT). The subsequent development and acceptance of MRI has been slower than that of CT. Recently there has been a dramatic improvement in MR image quality. MR imagers are now more widely available. Within 16 years CT, which represented such an enormous advance in diagnostic imaging when introduced in the early 1970s, is beginning to look vulnerable. The oncologist must now consider whether MR might replace CT.

The advantages of MRI over CT are most obvious when imaging the central nervous system. MRI is more sensitive than CT to changes in brain morphology and in turn more sensitive to the presence of tumour (Brant-Zawadski, 1988). The size and extent of primary brain tumours may be better appreciated on MRI, especially when a combination of T2 weighted and gadolinium DTPA contrast enhanced T1 (Gd-MR(T1)) images have been obtained. Gadolinium DTPA is injected intravenously and is distributed throughout the body in a manner analogous to that of iodinated contrast injected at CT examination. In the brain gadolinium DTPA will accumulate where the blood-brain barrier is deficient, and shortens the MR relaxation times directly in proportion to its local concentration. Tumours that enhance at post-contrast CT examination will also enhance at Gd-MR(T1) examination. The strengths and limitations of this technique are similar to those already seen at CT. Tumours may enhance strongly, and tumour may be separated from surrounding oedema or central necrosis. Unfortunately postoperative enhancement may be due to either residual/recurrent tumour, or the direct effect of surgery.

In the assessment of supratentorial tumours MRI is better than CT at demonstrating spread of tumour across the midline via the corpus callosum. T2 weighted MRI may demonstrate low-grade gliomas as areas of abnormal signal intensity, when the same lesions are invisible on CT as they exert no mass effect, exhibit no enhancement and are not associated with surrounding oedema. This is a very important diagnostic use of MRI (Pomeranz, 1989). CT in patients with low grade tumours may show only calcifications. The demonstration of abnormal signal on MRI examination may confirm the presence of low grade tumour, when questionable calcifications are seen at CT. The multiplanar capability of MRI is useful in assessing posterior fossa and extra-axial tumours, especially those arising in the pituitary fossa, neural foramina and dural spaces. Gd-DPTA contrast enhancement is important in the adequate depiction of these tumours (Berry *et al.*,1986).

Intracerebral metastases are recognised on CT mainly because of their multiplicity. Gd-DTPA MRI is slightly better than contrast enhanced CT at demonstrating small metastases. This may be useful particularly in the patient with an apparently solitary metastasis on CT imaging, who is being considered for local treatment, either resection or limited field irradiation. The demonstration of an additional lesions(s) would lead to a definite change in management. Gd-DPTA enhanced MRI may also demonstrate asymptomatic metastases in patients with normal CT brain scans (Cherryman *et al.*, 1989). Again this is useful if major changes in management would result from the demonstration of an unsuspected brain metastasis. There is also a suggestion that MR may help in the vexed question of diagnosing leptomeningeal metastatic disease (Davis *et al.*, 1987).

The spinal cord and its surroundings are an important site of disease spread, and an area often difficult to adequately image even with a combination of plain radiographs, myelography and CT scanning. MRI adds a new dimension to the imaging of this area (Haughton, 1988). The multiplanar capability of MR allows the spinal cord to be visualised in saggital, coronal and axial planes. Intraspinal tumours are recognised because of changes in morphology and tumour enhancement following Gd-DPTA injection. Extradural masses are easily appreciated and spinal cord compressions well seen (Williams *et al.*, 1989*a*). This allows for more appropriate treatment planning. Coronal MR sections are ideal at demonstrating paravertebral masses, dumbbell tumours and root compressions. MRI of the spine is the most important application of MRI in present oncological practice.

Outside the CNS MRI is of most value in assessing the soft-tissues. T1 weighted MR images are best at differentiating soft-tissues from fat. T2 weighted images allow the differentiation of normal from abnormal soft-tissues. This is a major advance on CT, as it is impossible to recognise soft-tissue dense tumour tissue within the muscles on CT until the shape of the muscle is altered. On T2 weighted MR images tumour spread into muscles may be visualised, even at an early stage when the muscle remains of normal shape. This is of most use in assessing the extent of soft-tissue tumours, including the extracranial head and neck. There are some caveats to the use of MRI in soft-tissue malignancy. First, MR is non-specific and it may not be possible to differentiate tumour from non-malignant causes of altered signal intensity. Second, the extent of tumours may be over-estimated as peritumoral oedema may appear of similar signal intensity and be incorrectly interpreted as tumour. MRI may be of value in the follow-up of tumours treated with chemotherapy and/or radiotherapy. Residual or recurrent tumour activity may be suspected from persistent or recurrent high T2 signal intensities.

MRI is also of value in assessing bone marrow. Metastatic involvement may be seen before the isotope scan is abnormal (Williams *et al.*, 1989*b*). The extent of marrow abnormality as demonstrated at MRI may also exceed that demonstrated on conventional radiographs, routine marrow aspirates/trephines and even specialised nuclear medicine techniques, such as mIBG scanning in metastatic neuroblastoma (Olliff *et al.*, 1989). MRI may also be useful in differentiating vertebral collapse due to osteoporosis from that associated with metastatic disease (Smoker *et al.*, 1987).

Received 21 August 1989.

The combination of sensitivity to marrow disease; spinal cord, root and nerve involvement; and soft tissue metastasis makes MR a useful tool in the investigation of pain or unexplained neurological change in the cancer patient. Clinical experience at the Royal Marsden (unpublished data) suggests that this will evolve into a major application of MR in oncology.

In the USA the impact of MRI on head and neck imaging has been dramatic. In the UK there are fewer MRI imagers available, and the value of the technique in this area is not widely appreciated. MRI scores over CT because of superior soft-tissue contrast, multiplanar imaging capability and excellent spatial resolution. Present opinion would consider MRI the modality of choice in evaluating the nasopharynx, paranasal sinuses, nasal cavity, floor of the mouth, tongue, oropharynx, parapharyngeal spaces, larynx salivary glands and neck (Som & Shapiro, 1989).

Abnormal lymph nodes may only be recognised on MRI once they have enlarged. Unfortunately MR cannot determine the cause of nodal enlargement or recognise the presence of nodal micrometastases (Dooms *et al.*, 1985).

In an oncological setting CT examination of the thorax is likely to remain superior to MRI. CT is the definitive method for the detection of small pulmonary metastases. In the mediastinum there is probably little significant difference between CT and MR. MR is subject to artifacts arising from respiratory, cardiac and other physiological movements. Artifacts may also be generated from flow phenomena.

In the abdomen the contribution of MR is also limited by these artifacts. The liver is the easiest organ to image and the present consensus is that MR of the liver complements CT and US examination. In general it is most cost effective to progress from simplest to most expensive, i.e. US, CT and only then MR.

MR is a still developing imaging technique, unlike CT which seems at present to have peaked. MR remains a more difficult examination than CT for both the patient and the radiologist. It is likely that some of the present difficulties associated with MR will be overcome. The use of MR contrast agents is a new and underexplored field; it is possible that the use of these agents might improve the specificity of the technique.

In present oncological practice MR of the spinal region and marrow is a revelation and has already altered clinical practice. MR will replace CT in brain, as well as extracranial head and neck imaging. In the remainder of the body CT remains supreme. MR is rightly limited to complementing CT examination of the soft-tissues. It is curious that we needed MR to appreciate just how useful body CT can be.

### References

AMBROSE, J. (1973). Computerized transverse axial scanning (tomography): Part 2: clinical applications. *Br. J. Radiol.*, **46**, 1023.

BERRY, I., BRANT-ZAWADSKI, M., OSAKI, L. *et al.* (1986). Gd-DPTA in clinical MR of the brain. II. Extraaxial lesions and normal structures. *Am. J. Neuroradiol.*, **7**, 789.

BRANT-ZAWADSKI, M. (1988). MR imaging of the brain. *Radiology*, **166**, 1.

CHERRYMAN, G.R., WILLIAMS, M.P., HUSBAND, J.E. *et al.* (1989). A prospective comparison of Gadolinium-enhanced magnetic resonance, T2 weighted magnetic resonance and contrast enhanced computed tomography in the detection and diagnosis of intracerebral metastases from small cell lung cancer. Presented at *Radiology '89*, Eastbourne, 6 May 1989.

DAVIS, P., FRIEDMAN, N., FRY, S. *et al.* (1987) Leptomeningeal metastasis. MR imaging. *Radiology*, **163**, 449.

DOOMS, G.C., HRICAK, H., MOSELEY, M.E. *et al.* (1985). Characterisation of lymphadenopathy by magnetic resonance relaxation times: preliminary results. *Radiology*, **155**, 691.

HOUGHTON, V.M. (1988). MR imaging of the spine. *Radiology*, **166**, 297.

HOUNSFIELD, G.N. (1973). Computerized transverse axial scanning (tomography). Part 1. Description of system. *Br. J. Radiol.*, **46**, 1016.

LAUTERBUR, P.C. (1973). Image formation by induced local interactions: examples using nuclear magnetic resonance. *Nature*, **242**, 190.

OLLIFF, J.F.C., MOYES, J.S.E., PINKERTON, C.R. *et al.* (1989). Magnetic resonance imaging of the marrow in patients with neuroblastoma: a comparison between magnetic resonance imaging MIBG marrow imaging and marrow aspirates. Presented at *Radiology '89*, Eastbourne, 5 May 1989.

POMERANZ, S.J. (1989). *Craniospinal Magnetic Resonance Imaging*. W.B. Saunders: Philadelphia.

SOM, P.M. & SHAPIRO, M.D. (1989). MRI of the head and neck. *Radiol. Clin. Nrth Am.*, **27**, 195.

SMOKER, W.R.K., GODERSKY, J.C., KNUTZEN, R.K. *et al.* (1987). The role of MR imaging in evaluating metastatic spinal disease. *Am. J. Roentgenol.*, **149**, 1241.

WILLIAMS, M.P., CHERRYMAN, G.R. & HUSBAND, J.E. (1989*a*). Magnetic resonance imaging in suspected metastatic spinal cord compression. *Clin. Radiol.*, **40**, 286.

WILLIAMS, M.P., CHERRYMAN, G.R. & HUSBAND, J.E. (1989*b*). Magnetic resonance imaging of spinal marrow in staging of patients with carcinoma of the breast. Presented at *Radiology '89*, Eastbourne, 6 May 1989.